# Sherry Wines

**Published:** 1890-06

**Authors:** 


### SHERRY WINES.

Sherry is one of the white wines of Spain. The name is derived from Xeres de la Frontera, a town in Andalusia, the frontier town of the Christians during the occupation of Cadiz by the Moors, from which port it is distant about sixteen miles.

It is the product of the vineyards of the triangular district formed by Xeres de la Frontera, Santa Maria, and Lucar de Barrameda, and watered by the rivers Guadalquivir and Gaudalete. Sherry, when pure, contains less free acid, and is not so stimulating as most wines, and agrees well with most constitutions. Dietetically speaking, it is the wine in most general use. It is also much used in pharmaceutical preparations, being used as an agent for the extraction of the medicinal properties of some drugs which require spirit for their solution. The wine merchants of Xeres never export their stock of the oldest and finest wines, and, in accordance with the price at which they are valued, so the wines are compounded. Thus, a butt of wine, said to be thirty or forty years old, will contain a portion of the vintage of several seasons, for, as the wine is drawn from the butt, it is replenished by contribution from the next in point of age and fineness, and so on to through the stock.

The Xeres grape has been planted in many places, but nowhere does it produce such fine wine as it does in its own native soil. Sherry is improved by being decanted not less than two hours before it is required for use. As a medicine, it is said to act beneficially upon a torpid liver, but in these days of ingenious adulteration, one is scarcely ever able to tell the genuine from the counterfeit, hence it is safest to rely upon our native dry wines for the sick, when prescribed by a physician.

